# Seronegative Catastrophic Antiphospholipid Syndrome: A Case Report of a Deadly Diagnosis

**DOI:** 10.7759/cureus.70785

**Published:** 2024-10-03

**Authors:** Nadim A Qadir, Michael Cargill, Hamza Choudhry, Oshin Rai, Parth Desai, Sanjay Lamsal, Pramod Reddy

**Affiliations:** 1 Internal Medicine, University of Florida College of Medicine - Jacksonville, Jacksonville, USA; 2 Radiology, University of Florida College of Medicine - Jacksonville, Jacksonville, USA

**Keywords:** acute limb ischemia, anti-phospholipid antibody syndrome (aps), catastrophic antiphospholipid syndrome (caps), left ventricular thrombus, multi-system thrombosis, seronegative catastrophic antiphospholipid syndrome

## Abstract

Catastrophic antiphospholipid syndrome (CAPS) is a rare autoimmune condition that causes diffuse hypercoagulability affecting multiple organ systems. Typically, CAPS is diagnosed via serological workup, clinical findings, or histopathological findings. However, patients with clinical features of CAPS have been found to be seronegative. This case report aims to inform clinicians of the growing array of CAPS serological markers and the need for further research into these markers to assess clinical viability. Here, we present a case of a 51-year-old female who presented with multi-system thrombosis and was diagnosed with seronegative CAPS (SN-CAPS). Given the significant mortality rate associated with CAPS, it is imperative that an early diagnosis is established, and treatment is initiated. Thus, continued serological research for CAPS is required to aid in this effort.

## Introduction

Antiphospholipid syndrome (APS) is an autoimmune condition that is characterized by arterial and venous thrombosis, recurrent fetal loss, and the presence of antiphospholipid antibodies [1,2]. The three main laboratory tests that are utilized to establish this diagnosis are lupus anticoagulant (LA), anti-beta-2 glycoprotein 1 antibodies, and anticardiolipin antibodies (aCL) [3]. With clinical manifestations highly suggestive of APS despite negative serum markers, the term seronegative APS was first suggested in 2003 [4]. There is also a subset of these patients who can go on to develop catastrophic antiphospholipid syndrome (CAPS), initially presented by Ronald Asherson in 1992 [5].

CAPS is defined by multi-system thrombosis in a short time frame and increased mortality than classic APS [5]. It is extremely rare to develop CAPS with approximately 1% of patients with APS being affected, and approximately only 500 reported cases to the CAPS registry [2]. Mortality rates for CAPS patients have been reported as high as 50% and despite guideline treatment of anticoagulation, steroid therapy, and plasma exchange (PLEX), mortality has only decreased to 37% [3,6]. Here, we present a case of a 51-year-old female who presented with findings suggestive of seronegative CAPS (SN-CAPS). This case report aims to discuss the difficulty in establishing this rare diagnosis, with the need for early recognition and treatment in such a deadly condition. Given the high mortality rate, further research on new antigen markers is imperative to assist in making the diagnosis for rapid treatment.

## Case presentation

A 51-year-old female with a past medical history of postural orthostatic tachycardia syndrome and hypothyroidism presented to the emergency department with bilateral lower extremity pain, peripheral cyanosis, and altered mental status. The family’s concern about worsening mental status prompted the patient’s hospital evaluation, and they noted the patient had pain in her lower legs bilaterally for months but over the past week, she felt significant pain with black discoloration of her toes. On the day of presentation to the hospital, the patient also developed a sudden onset facial rash and cyanosis of the nose. The patient had no recent sick contacts, travel, or history of rheumatological conditions, and was never known to be pregnant before.

**Figure 1:**
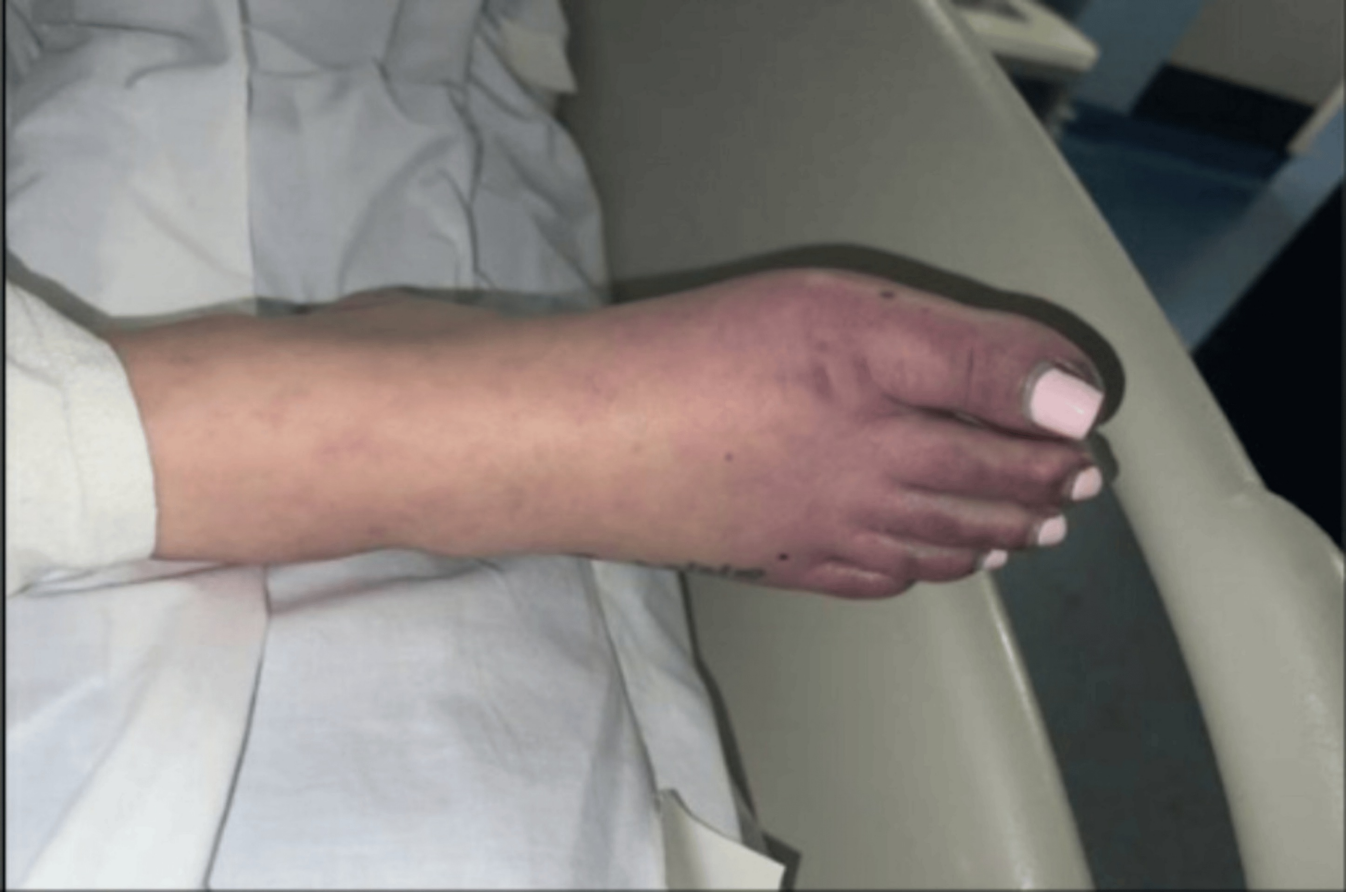
Blue discoloration of the right foot.

**Figure 2:**
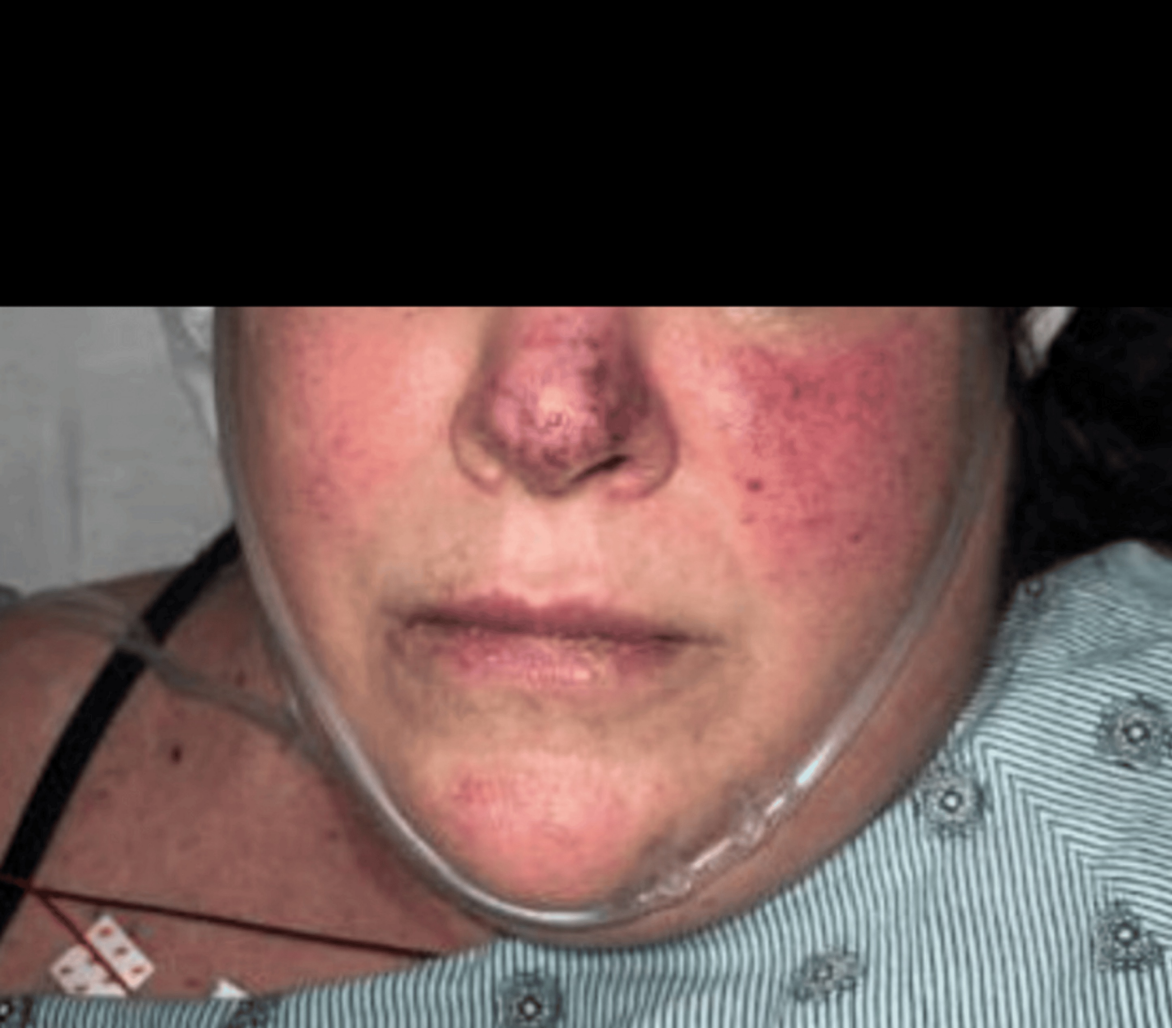
Bright red malar rash with blue discoloration of the nose.

Upon arrival, the patient was hypertensive with a blood pressure of 134/110 mmHg, otherwise, vitals were noted to be stable with an oxygen saturation of 99% on room air. The initial physical exam was remarkable for decreased sensation of the bilateral, cyanotic toes with 2+ pulses in the bilateral lower extremities (Figure 1). Also, a prominent erythematous malar rash with cyanosis of the nose was also identified (Figure 2). No signs of bleeding were noted on the physical exam.

The complete blood cell count revealed leukocytosis with a white blood cell count of 14.23 thou/cumm, normocytic anemia with hemoglobin at 10.3 g/dL, and thrombocytopenia with a platelet count of 70,000 per uL. A complete metabolic panel showed significant electrolyte derangements, as well as an acute renal failure with baseline creatinine, noted to be at 0.70 mg/dL (reference range: 0.51-0.95 mg/dL), acute liver failure, and a wide-anion gap metabolic acidosis (Table 1). Human immunodeficiency virus and hepatitis panel were found to be negative. A bedside cardiac ultrasound was significant for severely reduced ejection fraction (EF) with a prominent left ventricular (LV) thrombus visualized.

**Table 1:** Pertinent laboratory findings on initial presentation.

Parameters	Values	Reference Range
Sodium	131 mmol/L	136-145 mmol/L
Potassium	5.7 mmol/L	3.3-4.6 mmol/L
Chloride	99 mmol/L	98-107 mmol/L
Bicarbonate	9 mmol/L	21-29 mmol/L
Anion Gap	23 mmol/L	4-16 mmol/L
Blood Urea Nitrogen	78 mg/dL	6-22 mg/dL
Creatinine	5.78 mg/dL	0.51-0.95 mg/dL
Aspartate Aminotransferase (AST)	3,771 IU/L	14-33 IU/L
Alanine Aminotransferase (ALT)	4,522 IU/L	10-42 IU/L
N-Terminal Pro-Brain Natriuretic Peptide (NT-pro BNP)	37,527 PG/mL	0-125 PG/mL
D-Dimer	49,966 ng/mL	0-500 ng/mL
Platelet Count	70,000/µL	140,000-440,000/µL

**Figure 3:**
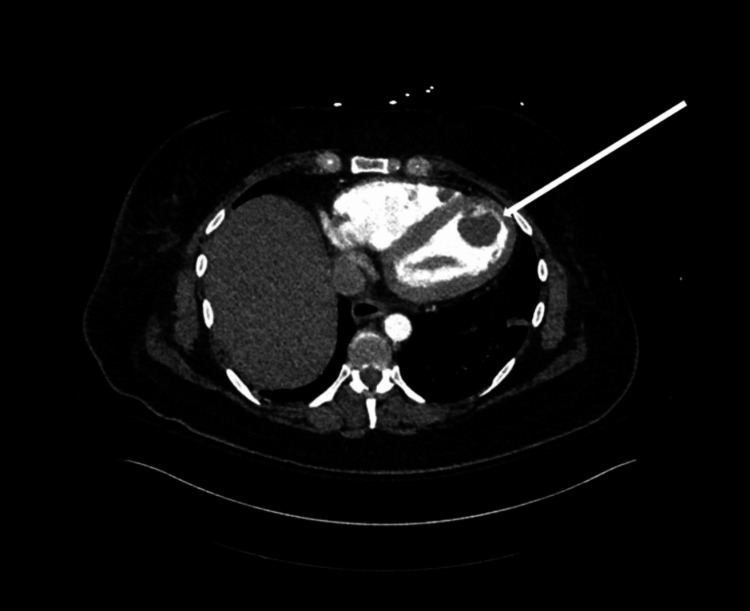
CT chest axial view demonstrating a filling defect within the left ventricle, consistent with intraventricular thrombus (white arrow).

**Figure 4:**
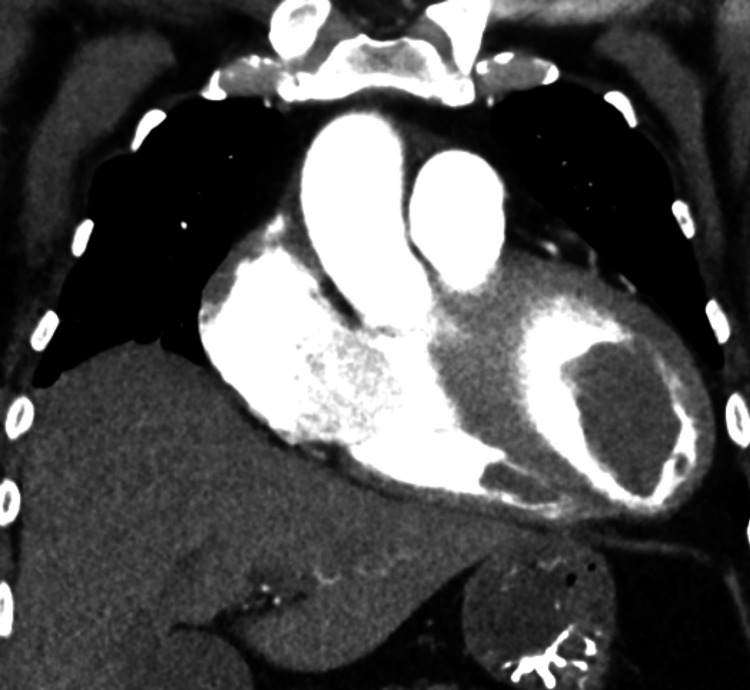
CT chest sagittal view of the left ventricular thrombus.

**Figure 5:**
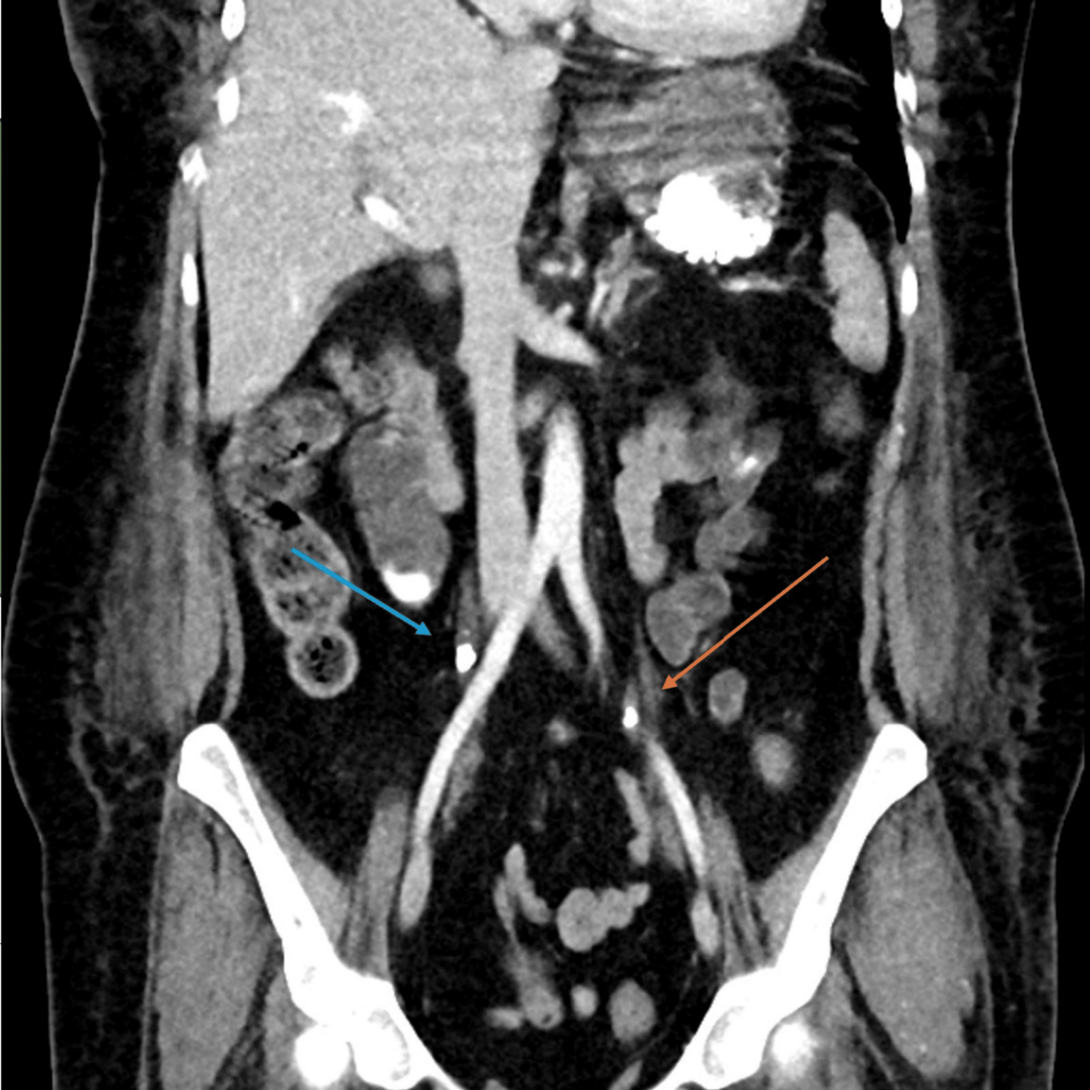
CT abdomen/pelvis sagittal view demonstrating bilateral nephrolithiasis, highlighting a 0.9cm stone in the left ureter (orange arrow) and a 1.0 cm stone in the right ureter (blue arrow) along with associated bilateral moderate hydronephrosis.

**Figure 6:**
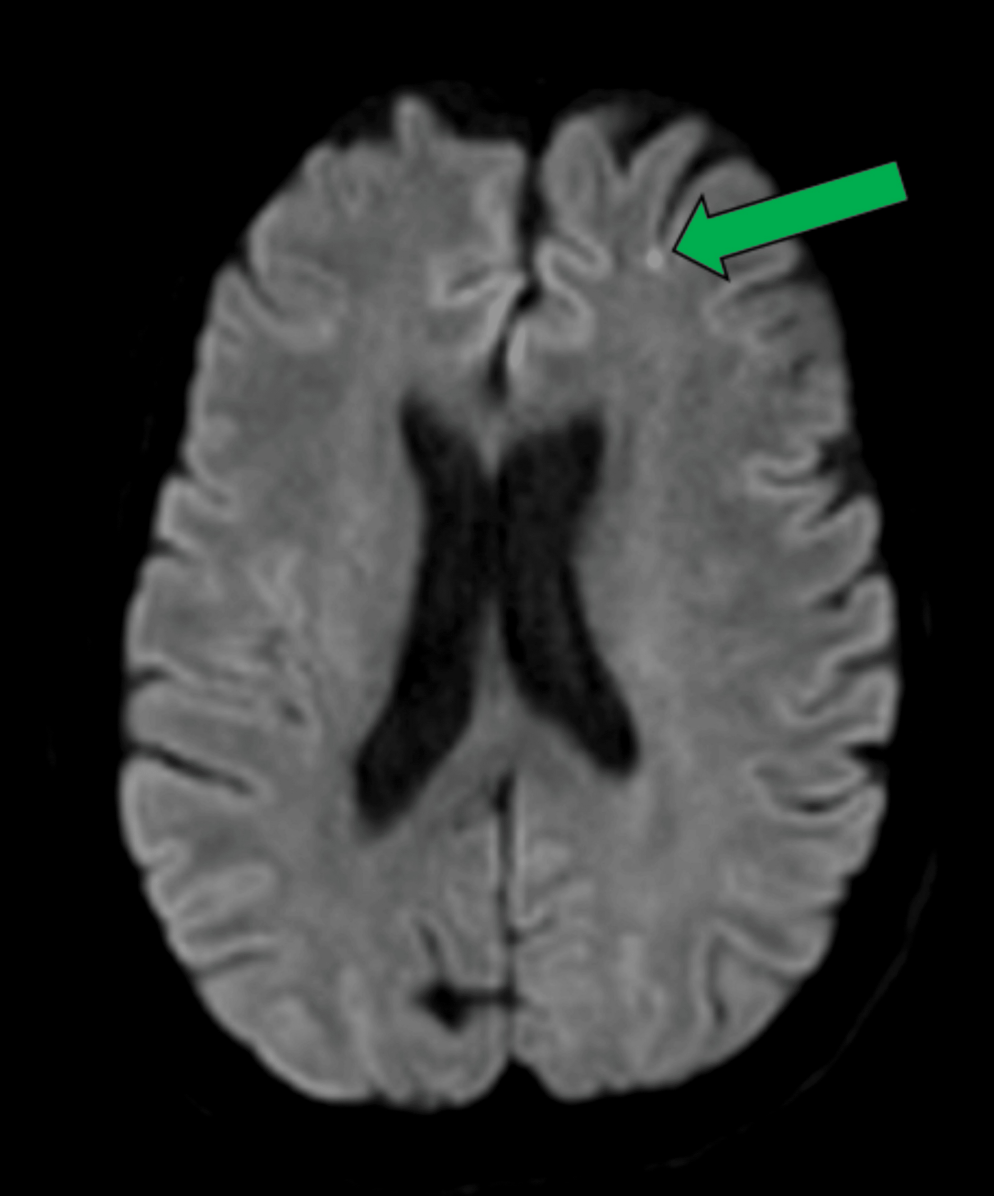
MRI axial DWI and ADC of the brain at the level of the lateral ventricles demonstrate a small focus of hyperintense DWI in the left frontal lobe (green arrow). These findings suggest a small subacute infarct. MRI: magnetic resonance imaging; DWI: diffusion-weighted imaging; ADC: apparent diffusion coefficient

Computed tomography (CT) of the chest, abdomen, and pelvis confirmed LV thrombus, with similar small filling defects found in the right ventricle, and bilateral nephrolithiasis with hydronephrosis (Figures 3-5). Also, pulmonary emboli in the right basilar segmental/subsegmental arteries were also seen. CT head was acutely unremarkable; however, due to the severity of the patient’s presentation, a magnetic resonance imaging (MRI) brain was pursued that showed a small punctate left frontal subcortical infarct (Figure 6). The patient was initiated on a heparin drip and transferred to the intensive care unit due to her critical condition. With the severity of the patient's presentation, including multiple system organ failure and hypercoagulable state, rheumatologic workup and antiphospholipid labs were ordered. An infectious workup was also obtained but found to be negative.

**Figure 7:**
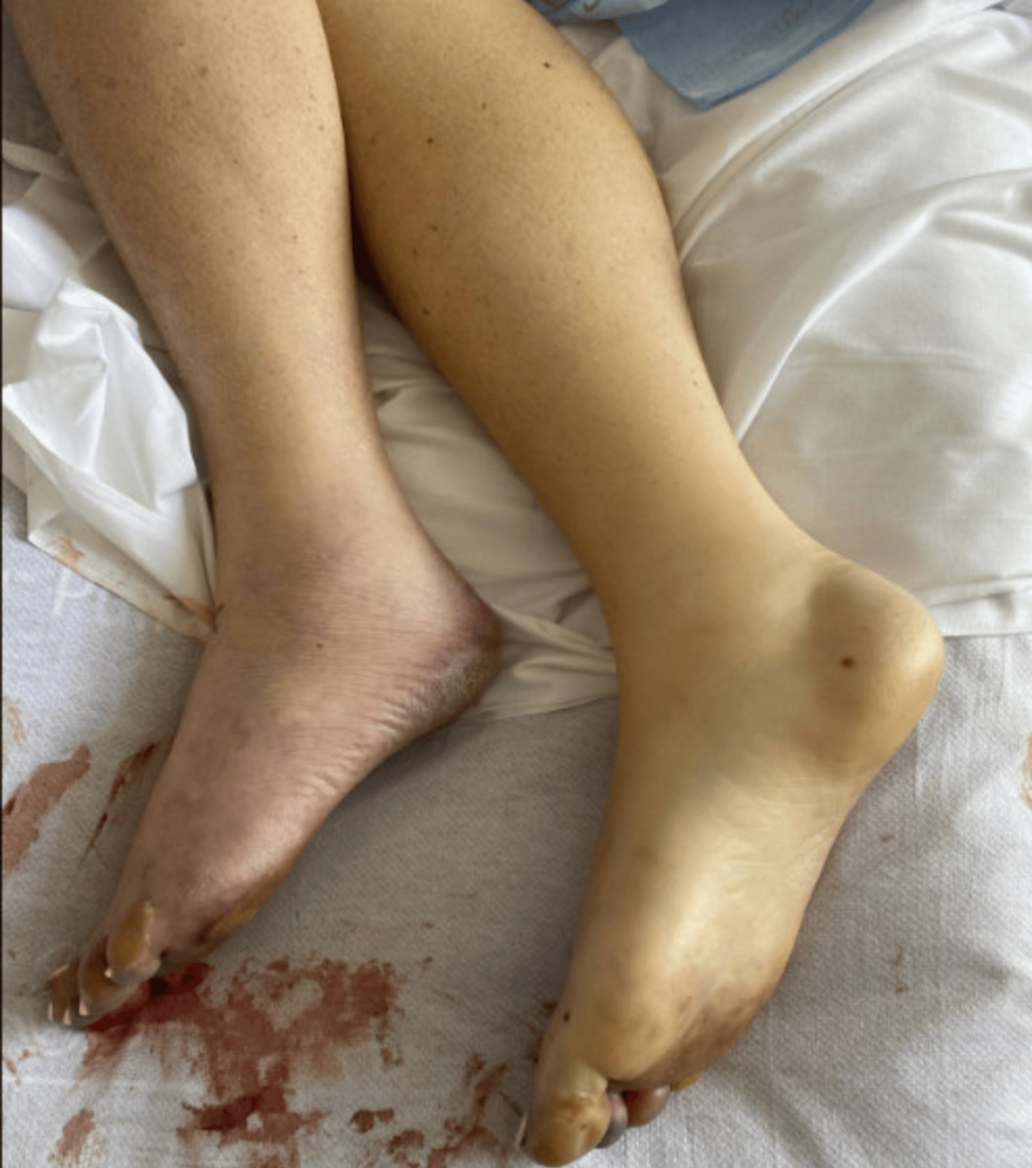
Pale bilateral lower extremities indicative of acute limb ischemia.

**Figure 8:**
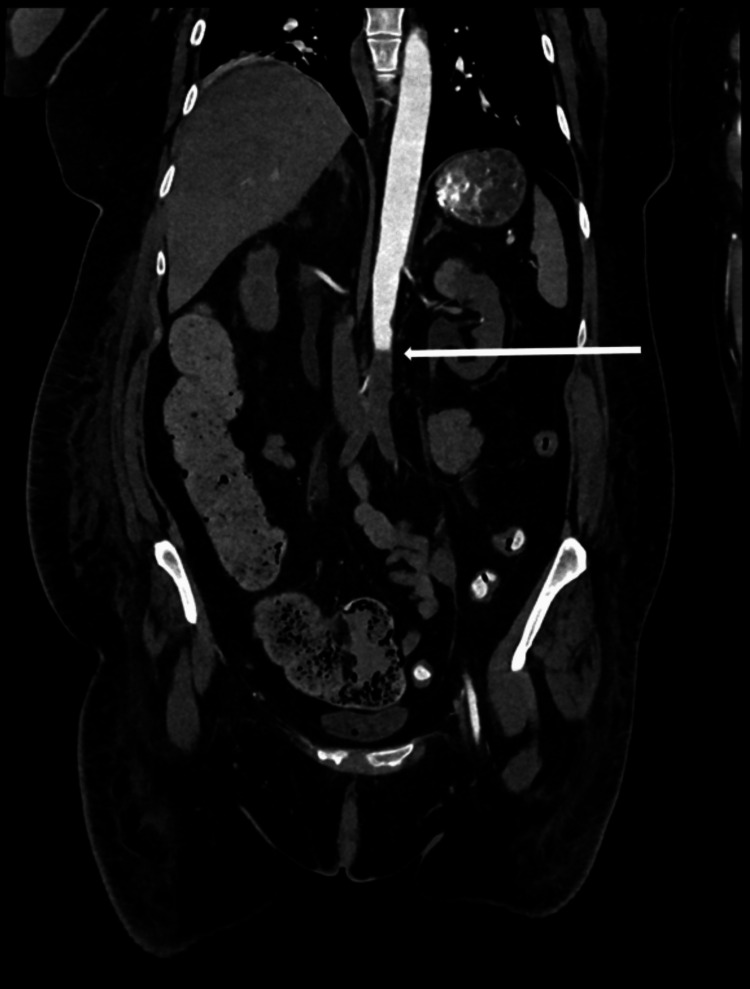
CT aorto-iliofemoral with runoff sagittal view showing an abrupt filling defect of the infrarenal aorta, consistent with acute intraluminal thrombus without distal reconstitution (white arrow).

Given the physical exam findings, initial lab findings, and imaging, the patient was initiated on intravenous steroids out of concern for CAPS. Methylprednisolone 1 mg/kg was initiated for five days. PLEX was also conducted for a total of three sessions. Importantly, intravenous immunoglobulin (IVIg) was not given during this admission. The patient’s mental status improved throughout her hospital course on CAPS treatment. Urology then placed bilateral renal stents due to the bilateral hydronephrosis. Then upon transitioning to warfarin, the patient noted extreme pain in the bilateral lower extremities, and on physical exam both legs were found to be pale with absent pulses (Figure 7). A bedside Doppler ultrasound was performed but could not detect pulses up to the proximal femoral artery. Subsequently, a CT aorto-iliofemoral scan with runoff revealed a saddle aortic thrombus (Figure 8). An emergent aortoiliac thrombectomy was then performed by vascular surgery and the patient was continued on a heparin drip, which was transitioned to warfarin without issue later in the hospital course.

The extensive rheumatologic workup was largely unremarkable, and antinuclear antibodies (ANA), anti-double stranded DNA, C-antineutrophil cytoplasmic antibodies (C-ANCA), beta-2 glycoprotein immunoglobulins, aCL antibodies, P-ANCA, anti-U1 ribonucleoprotein (RNP) antibody, anti-U3 RNP antibodies, atypical P-ANCA, anti-JO-1, anti-mitochondrial antibody, rheumatoid factor, CCP IgG antibodies, RNP antibodies, complement C1q, scleroderma antibody, Smith antibodies, anti-centromere B antibody, anti-histone antibodies, anti-glomerular basement membrane antibody, anti-SSA, and anti-SSB were negative. A hexagonal phase phospholipid panel was performed, revealing prolonged partial thromboplastin time (PTT) with a lupus anticoagulant time of 43.9 seconds and a significantly prolonged dilute Russell viper venom time (DRVVT) of 64.2 seconds; however, confirmatory repeat testing was negative later in the hospital course. Ancillary workup, including serum protein electrophoresis, was negative for monoclonal paraprotein. Serum protein C and protein S levels were also within normal limits.

Acute renal failure, hepatic failure, and metabolic derangements continued to improve throughout the hospital course with steroid therapy and plasmapheresis. Due to the severity of the patient’s initial presenting symptoms, with both arterial and venous thrombosis, as well as negative serum biomarkers, a diagnosis of SN-CAPS was made. Immunosuppression was not given, and a kidney biopsy was not done due to hydronephrosis. The patient was placed on a steroid taper and discharged to a rehabilitation center on warfarin indefinitely without any complications.

## Discussion

CAPS is a deadly complication of APS involving thrombosis in multiple organs over a short period. While APS is characterized by arterial and venous thromboembolic events, the criterion for CAPS includes (1) involvement of more than three organs, (2) symptom onset within one week, (3) histopathological confirmation of small vessel occlusion in at least one organ, and (4) laboratory confirmation of antiphospholipid antibodies. While fulfilling all four criteria classifies definite CAPS, probable CAPS can be classified if three criteria are present and the fourth is incompletely fulfilled [7]. SN-CAPS differs from CAPS in that patients do not demonstrate conventional serological markers of APS: anti-beta-2-glycoprotein 1, LA, or aCL by enzyme-linked immunoassay (ELISA) [8]. However, SN-CAPS still presents with the clinical and pathological findings of CAPS.

An analysis from an international CAPS registry database showed the following organs were most affected: 73% involvement of kidneys, 60% involvement of the lungs, 56% involvement of the brain, and 50% involvement of the heart [9]. The analysis found that in 65% of the cases, infection was the most common identifiable triggering factor [9]. Other triggers included surgery, malignancy, and pregnancy [9]. Our patient had involvement of the kidneys, brain, heart, liver, and skin; however, no clear trigger for the patient’s SN-CAPS was found.

The diagnosis of SN-CAPS was initially suspected in our patient due to the rapid-onset multi-organ system involvement, diffuse thrombosis, and extensive negative serological workup. Although thrombosis is common within small vessels, it also can be seen in large vessels as in our patient [10]. SN-CAPS is a difficult diagnosis to establish and other differential diagnoses such as disseminated intravascular coagulation (DIC), thrombotic storm, as well as other thrombotic pathologies should be considered. It is notable that although our patient presented with thrombocytopenia, fibrinogen level and PTT were normal. She had no signs of bleeding; therefore, DIC and other thrombotic microangiopathies were less likely. Additionally, our patient's positive response to CAPS management, including anticoagulation, plasmapheresis, and steroid therapy, suggested the possibility of SN-CAPS.

In regard to serological testing, one method of detecting lupus anticoagulants in APS is through dilute assays. As lupus anticoagulants are specifically directed against phospholipids to activate clotting factors, the presence of excess phospholipids in standard PT/PPT assays can neutralize the effects of these antiphospholipid antibodies. To counter this effect, dilute assays contain fewer phospholipids and can be used to assess each aspect of the coagulation cascade with higher sensitivity. Dilute assays include dilute prothrombin time, dilute aPTT, kaolin clotting time, and DRVVT. When one of these screening assays is prolonged, a hexagonal phospholipid neutralization test can be performed to see if the assay normalizes, thus demonstrating APS [11]. In our patient, the initial hexagonal phase phospholipid assay was initially positive; however, repeat confirmatory testing was found to be normal.

There are cases reported in medical literature of possible SN-CAPS, and theories have been postulated as to why serum markers continue to be negative in these patients [10,12-15]. These include the diagnosis made was inaccurate, antiphospholipid antibodies that were positive before are now negative, or the modern theory that the current range of tests for CAPS identification is inadequate [8]. Extra-criteria antibodies for CAPS have been identified which include anti-annexin V/annexin A5 resistance, vimentin/CL complex, phosphatidylethanolamine, and anti-prothrombin/phosphatidylserine [16]. Despite these findings, the clinical significance of these antibodies still requires further assessment. Also notable is the current testing for anti-beta-2-glycoprotein 1, LA, or aCL is routinely done by ELISA. However, in regard to SN-APS, there are studies indicating the presence of antiphospholipid antibodies by immunostaining on thin-layer chromatography not identified by ELISA [8].

There is no standardized treatment for CAPS due to its rarity and lack of clinical studies; however, expert opinion recommendations for initial treatment are triple therapy: anticoagulation, corticosteroids, and plasmapheresis or IVIg [17]. This treatment regimen for CAPS has the highest survival rate, though evidence for each treatment modality is low [3]. Other therapeutic options to be considered are cyclophosphamide in systemic lupus erythematosus (SLE) patients and rituximab, though these are not recommended as initial therapies for CAPS [18]. In this case, unfractionated heparin was the first treatment modality to be started, steroids and plasmapheresis were completed subsequently as suspicion for the diagnosis of CAPS increased. However, no IVIg or other alternative treatment modalities were initiated in this case as the patient improved throughout the hospital course.

## Conclusions

With the recognized difficulty in establishing a diagnosis of SN-CAPS, if the clinical suspicion for CAPS is high enough as in our patient, we urge clinicians to consider immediate treatment. With the very high mortality rate associated with CAPS, it is vital that treatment be initiated rapidly for improved patient outcomes. The purpose of this paper is to highlight the knowledge gap that still remains in establishing this diagnosis, and although extra-antibodies have been identified in APS patients, the clinical significance still remains unclear. Further studies and research testing are imperative for patients diagnosed with SN-CAPS.
